# Associations between the Genetic Heritability of Dyslipidemia and Dietary Patterns in Korean Adults Based on Sex Differences

**DOI:** 10.3390/nu15204385

**Published:** 2023-10-16

**Authors:** Sei Kim, Hye Kyung Jeon, Gyeonghee Lee, Youbin Kim, Hae Young Yoo

**Affiliations:** 1 Graduate School, Chung-Ang University, Seoul 06974, Republic of Korea; seikim98@gmail.com (S.K.); lktina0915@gmail.com (G.L.); gkgkg1101@naver.com (Y.K.); 2 Department of Nursing, Ansan University, Ansan 15328, Republic of Korea; musenike@naver.com; 3 Department of Nursing, Chung-Ang University, Seoul 06974, Republic of Korea

**Keywords:** dyslipidemia, diet patterns, genetic risk score, lipids

## Abstract

Dyslipidemia can be defined as an abnormality in serum lipid levels that is substantially linked to genetic variations and lifestyle factors, such as diet patterns, and has distinct sex-specific characteristics. We aimed to elucidate the genetic impact of dyslipidemia according to sex and explore the associations between genetic variants and dietary patterns in large-scale population-based cohorts. After performing genome-wide association studies (GWASs) in male, female, and entire cohorts, significant single nucleotide polymorphisms (SNPs) were identified in the three groups, and genetic risk scores (GRSs) were calculated by summing the risk alleles from the selected SNPs. After adjusting for confounding variables, the risk of dyslipidemia was 2.013-fold and 2.535-fold higher in the 3rd quartile GRS group in the male and female cohorts, respectively, than in the 1st quartile GRS group. While instant noodle and soft drink intake were significantly associated with GRS related to hyperlipidemia in male cohorts, coffee consumption was substantially related to GRS related to hyperlipidemia in female cohorts. Considering the influence of genetic factors and dietary patterns, the findings of this study suggest the potential for implementing sex-specific strategic interventions to avoid dyslipidemia.

## 1. Introduction

Dyslipidemia is characterized by imbalanced lipid concentrations in the blood, which include high levels of total cholesterol (TC), triglycerides (TGs), and low-density lipoprotein cholesterol (LDL-C), or low levels of high-density lipoprotein cholesterol (HDL-C) [1]. Dyslipidemia is a chronic metabolic disorder that can cause atherosclerosis, ultimately leading to cardiovascular diseases (CVDs) [2]. CVD is a major health problem worldwide due to its high morbidity and mortality, accounting for 32% of all global deaths [3,4]. Early detection, prevention, and appropriate management of CVDs are essential to reduce personal and social burdens and costs; thus, dyslipidemia is an important modifiable risk factor [5]. Regarding CVDs, there are well-described differences between males and females because sex hormones, sex-specific gene expression, and epigenetic modifiers interact with the disease throughout an individual’s lifespan [6]. Dyslipidemia has distinct characteristics based on sex, not only in terms of epidemiological and pathophysiological aspects but also in genetic traits [7,8,9]. Several studies have discovered that males and females show dissimilar lipid metabolism, such as a greater percentage of men than women with elevated TG concentration in the blood, or the tendency for middle-aged women to have higher levels of HDL-C than men [10,11,12]. Interestingly, a single nucleotide polymorphism (SNP) in *APOA5* was associated with dyslipidemia only in male subjects [13].

Nutrition and diet strongly impact the development of dyslipidemia, and it is well known that food choices are crucial in its prevention and management [14]. Consuming a diet high in fructose, trans-fatty acids, or sugar-sweetened beverages can alter blood lipid levels to an abnormal range. In addition, some dietary habits, such as a high intake of omega-3-rich foods, nuts, or dietary fiber, are known to help maintain lipid levels within the range of non-dyslipidemia [15,16,17]. Korean cuisine is known for its unique features, and is characterized by fermented foods, soup with spicy seasonings, and high pork consumption [18,19]. In addition, a significant proportion of Koreans have shifted to the use and consumption of recipes from other countries [20]. The substantial salt intake of Koreans is also notable, being approximately 1.87 times higher than recommended by the World Health Organization (WHO) [21]. Given that various Korean diets may affect dyslipidemia, few studies have been conducted [22,23].

The genetic risk score (GRS) is a small score based on specific alleles at SNPs that can indicate inherited heritability in individuals from a genome-wide association study (GWAS) [24]. Since GRS approaches have recently attracted considerable interest, this score has been used to predict an individual’s risk of developing a particular disease or trait, as well as to develop new interventions by dissecting the interaction of genes and environmental factors [24,25]. To date, the GRS has been used in dyslipidemia and CVD risk studies and shows great risk-predictive value; however, more studies are needed with regard to ethnicity and sex [26]. Although the GRS of dyslipidemia has cumulative effects on blood lipid levels in Koreans [27], its interaction with diet has not been fully elucidated. 

Gene–diet interactions can modify the outbreak and severity of dyslipidemia and propose life-course prevention in early life [28]. To the best of our knowledge, no study has investigated whether Korean dietary patterns are associated with sex-specific genetic variants of dyslipidemia. Therefore, we aimed to clarify genetic heritability based on GRS and examine the associations between dietary patterns and sex-specific genetic variants related to dyslipidemia in a Korean cohort.

## 2. Materials and Methods

### 2.1. Participants

**Figure 1:**
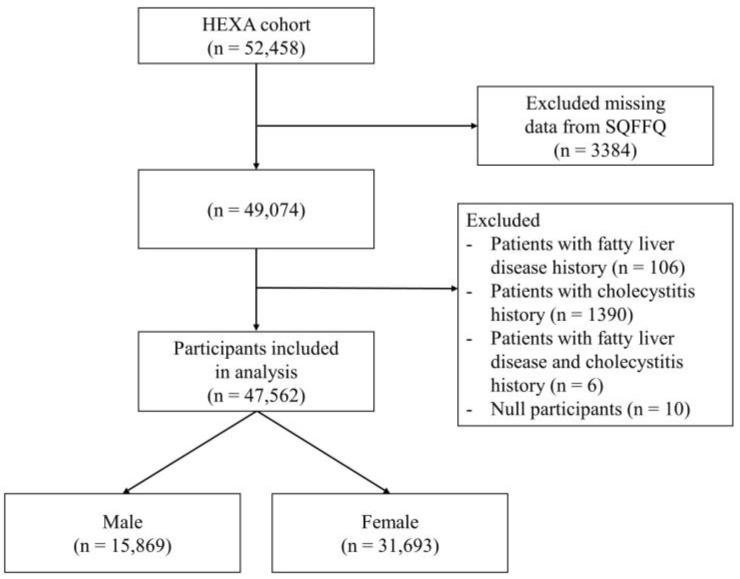
Flowchart of the study participants. SQFFQ, semi-quantitative food frequency questionnaire.

The Korean Genome and Epidemiology Study (KoGES), conducted by the Korea National Institutes of Health, recruited health examinee (HEXA) cohorts of Korean individuals aged 41–85 years living in several cities, which is a large prospective population-based cohort. Participants visited designated medical institutions to undergo health screenings and complete surveys to determine their health status and lifestyle [29]. A total of 52,458 individuals had available epidemiological and genomic data. We excluded individuals with missing data from the analysis. The exclusion criteria were subjects who had been diagnosed with fatty liver or cholecystitis [30,31,32]. After exclusion, 47,562 participants remained, as shown in Figure 1. 

### 2.2. Biochemical Measurements

Fasting serum lipid levels were measured using an automated chemistry analyzer (ADIVA 1650, Siemens, Tarrytown, NY, USA). Detailed methods have been described previously [29]. We identified dyslipidemia cases based on whether subjects met any of the following criteria: TC ≥ 240 mg/dL, LDL-C ≥ 160 mg/dL, TGs ≥ 200 mg/dL, or HDL-C < 40 mg/dL, according to the Korean Society of Lipid and Arteriosclerosis Korean Guidelines for the management of dyslipidemia [33]. 

### 2.3. Genotyping, Quality Control, GWASs, and Calculating GRS

Genomic DNA samples were genotyped by the Korean Biobank Array, also called K-Chip [34]. Genotyping was performed to determine the genotypes of 7,079,946 SNPs in 58,700 individuals. SNP quality control (QC) was conducted to purify the genomic data and analyze 47,562 of the 58,700 subjects from the HEXA study included in the K-chip. Finally, 5,417,091 SNPs were analyzed after QC by applying a minor allele frequency (MAF) < 0.05, SNP call rate < 0.95, and Hardy–Weinberg equilibrium (HWE) *p*-value < 1 × 10^−6^. Data purification and SNP detection were performed using Plink Ver 1.9 at http://pngu.mgh.harvard.edu/purecell/plink/ (accessed on 4 May 2023).

The GWASs were performed for the entire cohort, and for male and female participants separately using logistic regression analysis. Age, sex, and body mass index (BMI) were used as covariates. Significant SNPs were detected in each of the three groups using functional mapping and annotation of genome-wide association studies (FUMA-GWASs) at https://fuma.ctglab.nl/ (accessed on 4 May 2023) [35]. 

The GRS was calculated by summing the scores of 0, 1, or 2 points for each significant SNP, depending on the number of risk alleles of all lead SNPs from the GWASs, using the –score function in Plink Ver 1.9.

Following the calculation of the GRSs, the scores were classified into three subgroups: low scores in the 1st quartile, intermediate scores in the 2nd quartile, and high scores in the 3rd quartile, based on a previous study.

### 2.4. Dietary Patterns

Dietary pattern data were gathered using a semi-quantitative food frequency questionnaire (SQFFQ) developed and validated by the Korea Centers for Disease Control and Prevention [36]. Among the 106 food items in the SQFFQ, the frequency of the intake of pork belly, beef, intestines, sausage, chicken, soup, instant noodles, snacks, soda, and coffee was used for analysis in this study. We used ≤70th percentile as the cut-off for dividing into low and high intake frequency groups, based on answers of never, once a month, 2–3 times per month, 1–2 times a week, 3–4 times a week, 5–6 times a week, once a day, twice a day, and ≥3 times per day [37].

### 2.5. Statistical Analysis

All statistical analyses were conducted using SPSS Ver 26.0 (IBM, New York, NY, USA), and figures were generated using R Ver 4.3.0 (R Foundation for Statistical Computing, Vienna, Austria) and Origin Pro Ver 8 (OriginLab Corporation, Northampton, MA, USA). 

The demographic characteristics of the HEXA cohort were described and divided into groups according to the presence or absence of dyslipidemia in males, females, and the entire cohort. Categorical variables are expressed as numbers and frequencies, whereas continuous variables are expressed as means and standard errors of the mean (SEM). Statistical differences in frequency or mean were assessed using Student’s *t*-test, chi-square test, and one-way analysis of variance (ANOVA). Post hoc Scheffé tests were used for multiple comparisons among the GRS quartiles in each male and female cohort. We conducted a subgroup analysis by age, dividing the cohorts into middle-aged (41–60 years) and elderly (61–85 years).

To evaluate the effect of GRS on dyslipidemia, the adjusted odds ratio (OR) and 95% confidence interval (95% CI) for dyslipidemia incidence were calculated with the 1st quartile as the reference category, separately for males, females, and the entire cohort.

Participants were categorized into high- and low-intake groups based on their dietary patterns, as described above. The interaction between GRS quartiles and dietary patterns was analyzed using binary multivariate logistic regression after adjusting for covariates, including age, sex, and BMI. The adjusted OR and 95% CI were calculated using the 1st quartile as the reference. Statistical significance was set at *p*-value < 0.05.

## 3. Results

### 3.1. Demographic Characteristics of Study Participants

The demographic characteristics and dietary patterns of the participants are presented in Table 1 and Table 2. Although the average age of individuals with dyslipidemia was lower than that of individuals without dyslipidemia in the male cohort, the average age of individuals with dyslipidemia was higher than that of individuals without dyslipidemia in females. BMI was significantly higher in the dyslipidemia group than in the non-dyslipidemia group in both the male and female cohorts, as well as in the entire cohort (Supplementary Table S1). Among the blood lipid levels, TC, TG, and LDL-C concentrations were significantly higher in dyslipidemia subjects than in non-dyslipidemia subjects in the male and female cohorts. The HDL-C level in dyslipidemia subjects was lower than that in non-dyslipidemia subjects in all cohorts. There was a higher percentage of current smokers among men and women with dyslipidemia than among those without dyslipidemia. In the female cohort, current alcohol drinkers were significantly fewer among individuals with dyslipidemia than among those without. Only male participants with dyslipidemia had a significantly wider waist circumference than male participants without dyslipidemia. Among both males and females, those with dyslipidemia had wider hip circumferences than those without dyslipidemia. Individuals with dyslipidemia had a significantly higher GRS than those without dyslipidemia in both male and female cohorts (Table 1).

### 3.2. Significant SNPs Related to Dyslipidemia

In the male cohort, 16 SNPs were identified by GWAS (*p* < 5 × 10^−8^) (Table 3A). Among these, rs662799, located in the *APOA5* gene, was the most significant (*p* = 2.11 × 10^−57^). In rs662799, individuals who carried A:G and G:G alleles showed a 1.506-fold and 2.472-fold higher risk of dyslipidemia incidence compared to those who carried A:A alleles, respectively (Table 4).

In the female cohort, GWAS analysis revealed 20 significant SNPs, and the rs651821 variant located in the *APOA5* gene was the most significant (*p* = 1.10 × 10^−77^). Subjects with T:C and C:C alleles of rs651821 had 1.362 and 2.212 times higher odds of dyslipidemia incidence, respectively.

Meanwhile, 25 SNPs were identified in the entire cohort. The most significant variant was rs651821 in the *APOA5* gene (*p* = 2.491 × 10^−132^), which was present as the most significant variant in the female cohort but not found in the male cohort (Supplementary Table S3).

### 3.3. OR of Dyslipidemia Incidence by GRS Quartiles

**Figure 2:**
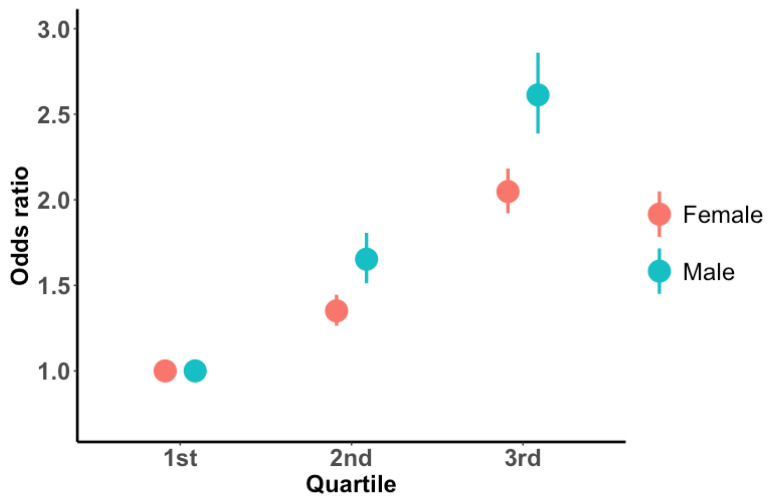
Adjusted OR plot of dyslipidemia incidence by GRS quartiles. GRS was calculated by summing the number of risk alleles in significant SNPs and was classified into 1st to 3rd quartiles based on increasing values. The logistic regression was conducted with the covariates, including age and BMI. The 1st quartile of dyslipidemia GRS was set as a reference group in each of the male and female cohorts.

Females in the 2nd quartile had a 1.339 times higher risk of dyslipidemia than those in the 1st quartile, whereas males in the 2nd quartile had a 1.627 times greater risk of dyslipidemia than those in the 1st quartile. Female individuals in the 3rd quartile group had a 2.013 times higher risk of dyslipidemia than females in the 1st quartile of the GRSs, and male individuals in the 3rd quartile group had a 2.535 times higher risk of dyslipidemia than males in the 1st quartile (Figure 2).

### 3.4. Blood Lipid Concentrations and Biochemical Parameters Stratified by GRS Quartiles

**Figure 3:**
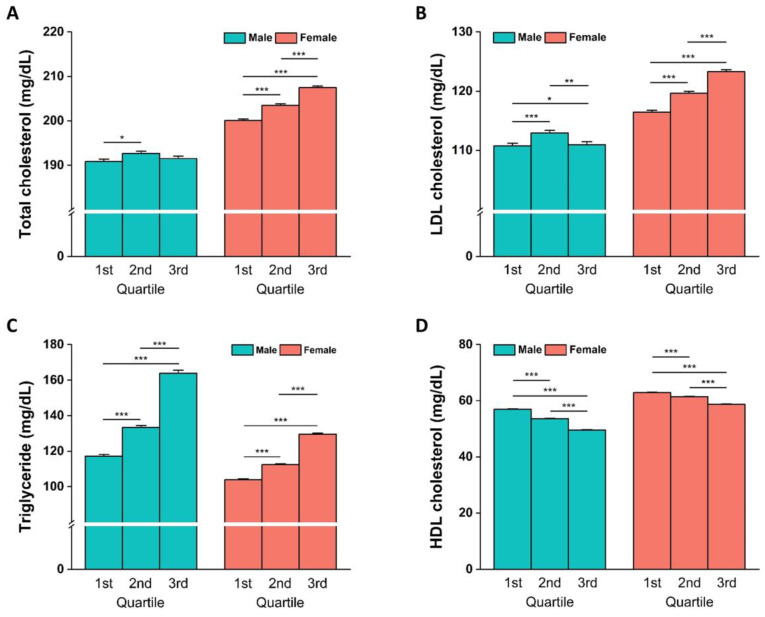
Comparison of blood lipid concentration by GRS quartiles. Means and SEM of total cholesterol (TC) (**A**), low-density lipoprotein cholesterol (LDL-C) (**B**), triglycerides (TGs) (**C**), and high-density lipoprotein cholesterol (HDL-C) (**D**) in the GRS quartiles by sex are presented. One-way ANOVA with the post hoc Scheffé test was conducted. Significant differences between GRS quartiles within male or female cohorts are represented by * *p* < 0.05, ** *p* < 0.01, and *** *p* < 0.001.

Males in the 1st and 3rd quartiles did not show different TC levels, and the LDL-C concentration in the 3rd quartile was lower than that in the 2nd quartile. In the female cohort, the blood concentrations of TC and LDL-C increased in the higher quartiles (Figure 3A,B). Serum TG concentration in the higher quartile was higher than that in the lower quartile in both cohorts (Figure 3C). HDL cholesterol levels were significantly lower in the lower quartile group than in the higher quartile group in both males and females (Figure 3D). The gamma-glutamyl transferase (λ-GTP) levels were significantly higher in 3rd quartile male and female individuals than those of males and females in the 1st quartile. Only female subjects in each quartile showed different albumin, aspartate aminotransferase, and creatinine concentrations in their blood, whereas quartiles from the male and female cohorts showed differences in alkaline phosphatase levels (Table 5). In middle-aged males and females, however, there were no significant differences in biochemical parameters stratified by GRS quartiles (Supplementary Table S4). Among the elderly males, λ-GTP levels were significantly higher in the 3rd quartile than those in the 1st quartile. Blood calcium levels in elderly males were significantly higher in the 3rd quartile than those in the 1st and 2nd quartile. In addition, it was found that the λ-GTP and ALP levels were significantly higher in the 2nd quartile than those in the 1st quartile of elderly females (Supplementary Table S5).

### 3.5. Interaction between GRS and Dietary Patterns

Table 6 shows the associations between the GRS for dyslipidemia and dietary patterns in male and female cohorts. In the male cohort, instant noodle intake and soft drink intake were significantly related to dyslipidemia risk in the higher quartile of the GRSs (*p* = 0.028 and *p* = 0.030, respectively). The higher GRS quartiles of males exhibited a greater OR compared to the lower GRS quartiles when considering the interaction with instant noodles. Soft drink intake also interacted with higher GRS quartiles for dyslipidemia risk in males and the entire cohort (Supplementary Table S6). In the entire cohort, there was an interaction between GRS and the consumption of soft drinks, affecting dyslipidemia (Supplementary Table S6, *p* < 0.001). Interestingly, coffee consumption was significantly associated with GRS for dyslipidemia only in the female cohort (*p* = 0.038). The OR increased with an increase in GRS quartiles in females. Except for the intake of instant noodles, soft drinks, and coffee, the other dietary patterns did not show any interaction with GRS for dyslipidemia risk.

## 4. Discussion

In the present study, we identified sex differences in genetic susceptibility to dyslipidemia and evaluated whether the GRS is associated with blood lipid concentrations in a population-based Korean cohort. We also confirmed the interaction between the GRS and dietary patterns in relation to dyslipidemia risk by sex. The GRSs were calculated from 16 and 20 SNPs related to dyslipidemia in the male and female cohorts, respectively. In both cohorts, the risk of dyslipidemia was more than two times higher in the higher GRS group than in the lower GRS group. Furthermore, sex differences in dietary patterns affected dyslipidemia and genetic heritability. Both instant noodle and soft drink intake were substantially linked with the risk of dyslipidemia in male participants, whereas coffee consumption was significantly associated with the risk of dyslipidemia in female participants.

Sex differences in the prevalence and pathophysiology of dyslipidemia are well established [7,8]; however, there is a gap in the literature regarding the genetic architecture of dyslipidemia. Our findings on sex-specific SNPs reveal evidence that genetic susceptibility to dyslipidemia differs by sex because most of the discovered SNPs did not overlap. Notably, the SNPs from the female cohort were located in tribbles pseudo kinase 1 (TRIB1), alpha 1-3-N-acetylgalactosaminyltransferase and alpha 1-3-galactosyltransferase (ABO), hemoglobin subunit gamma 2 (HBG2), SIK family kinase 3 (SIK3), and translocase of outer mitochondrial membrane 40 (TOMM40), whereas the SNPs from the male cohort were located in lipoprotein lipase (LPL), protein tyrosine phosphatase receptor type D (PTPRD), chloride voltage-gated channel 7 (CLCN7), leucine-rich repeat-containing G-protein-coupled receptor 5 (LGR5), cytochrome P450 family 2 subfamily D member 6 (CYP2D6), and cholesteryl ester transfer protein (CETP). While the apolipoprotein 5 (APOA5) gene is considered integral to lipid homeostasis [38], two different polymorphisms of this gene, rs662799 A > G and rs651821 T > C, were associated with a significant increase in the incidence of dyslipidemia in male and female subjects, respectively. Each GRS derived from male- and female-specific SNPs had different tendencies of TC and LDL-C concentration levels in the blood, which may be related to the fact that females have greater genetic heritability of cholesterol and LDL-C compared with males [39]. The GRSs from the male and female cohorts interacted with TG and HDL-C levels in opposite directions. Increased TG level by PRS is causally linked to coronary artery disease and has already been reported in another cohort [40]. However, to the best of our knowledge, decreasing HDL-C by GRS quartiles related to dyslipidemia is a new discovery. 

The results from the male participants confirmed that a high intake of instant noodles had a significant impact on the genetic risk of dyslipidemia. While instant noodles are primarily composed of saturated fats and carbohydrates, they also contain very few proteins [41]. In addition, instant noodles are not recommended for consumption by patients with diabetes and metabolic syndrome because hydrogen peroxide (H_2_O_2_), a type of reactive oxygen species, inhibits blood sugar control [42,43]. Furthermore, in a previous study confirming the relationship between instant noodle intake and lipid concentration levels in Koreans, instant noodle intake was found to have a negative effect on blood serum levels [44]. In 3397 college students, consuming instant noodles more than three times a week increased the risk of developing hypertriglyceridemia by 2.6 times, compared to eating them less than once a month [45]. This is the first study to identify a sex difference in the association between instant noodle intake and the genetic heritability of dyslipidemia; as such, the precise mechanism underlying this sex disparity should be explored in future studies. Several previous studies have identified sex differences in the associations between instant noodle consumption and risk factors for chronic diseases, such as metabolic syndrome and hypertension [46,47], but the effects of genetic variants by sex have not been considered. Therefore, individuals with these genetic characteristics should refrain from eating instant noodles, which are currently enjoyed by many Koreans.

Analysis of female participants confirmed that high coffee intake had a statistically significant effect on the genetic risk of dyslipidemia. Coffee is a complex mixture of various compounds, including a diterpene alcohol, which increases blood cholesterol levels [48]. Moreover, previous studies have shown that coffee alters blood lipid concentrations to within abnormal ranges [49]. In a study investigating 1017 Westerners, coffee consumption substantially contributed to an increase in TC, LDL-C, and TG levels, and lipid levels varied significantly with the frequency of consumption, while, on the other hand, it has been reported that genetic variations within the *ADORA* gene family increase the risk of dyslipidemia in Korean adults who consume coffee [50,51]. In addition, rs662799 of *APOA5*, one of the key SNPs used for GRS calculation in this study, had a significant association between human complex diseases and coffee consumption in a previous study in Japan. Our findings are very consistent with this previous study that showed a close link between SNPs, which cause cancer, diabetes, and cardiovascular disease in the Japanese population, and coffee consumption. Moreover, our research is highly reliable in that it was conducted with subjects of the same ethnicity [52]. Despite these concerns, coffee consumption has rapidly increased in South Korea, with adults consuming an average of 353 cups annually [53]. Moreover, it is of grave concern that the majority of coffee consumed by Koreans consists of coffee mixes, coffee beverages, and instant coffee [54]. In contrast to coffee beans, mixed coffee, coffee beverages, and instant coffee contain a substantial quantity of sugar, which exacerbates the negative effects of cardiovascular disease [54,55,56]. Considering these points, women at high genetic risk for dyslipidemia should regulate their coffee consumption, and dietary guidelines for coffee intake should be developed to prevent and manage dyslipidemia.

Soft drink intake had a statistically significant effect on genetic predisposition to dyslipidemia in the entire cohort and male participants in this study. Soft drinks are a major source of sugar that affects serum lipid concentrations. Soft drink consumption reduces HDL-C and increases LDL-C and TG levels [57,58]. In addition, a previous study found that soft drink intake increased the incidence of obesity and metabolic syndrome [59]. In particular, there was a strong association between obesity, low HDL-C levels, and metabolic syndrome in middle-aged Korean men [60]. Several previous studies have confirmed sex differences in soft drink consumption and cardiovascular diseases [61,62]; however, to our knowledge, no study has considered the genetic predisposition to dyslipidemia. Therefore, it is crucial for individuals with genetic polymorphisms associated with dyslipidemia to avoid consuming soft drinks as much as possible to reduce the risk of dyslipidemia. Furthermore, as the gut microbiota is a causal factor of dyslipidemia and the microbiota-dependent mechanism of dietary sugar elaborates the interaction with metabolism, future studies should integrate gut microbiota with dietary patterns to comprehensively understand the multifaceted interactions with dyslipidemia [63,64].

Meat consumption is known to increase the risk of cardiovascular diseases owing to the unfavorable effects of saturated fat contained in red meat [65]. In addition, compared to unprocessed red meat, processed meat contains excessive salt, which can negatively affect blood lipid levels. Unexpectedly, no significant associations were found between the GRS for dyslipidemia and meat consumption, such as pork belly, intestines, beef, and sausages, in the present study. This also conflicts with the results of a recent study conducted on Korean HEXA cohorts [66], which showed that increased red meat consumption was associated with an increased risk of dyslipidemia; however, genetic predisposition was not considered. Since dyslipidemia is influenced by a complex interaction between genetic and environmental factors [7,8,9], this study confirmed the association between the GRS of dyslipidemia and meat consumption. 

The present study has a number of strengths. First, this is the first study to examine the interaction between dietary patterns and sex-specific GRS for dyslipidemia in a large prospective cohort of Koreans. Moreover, dietary factors, including the intake of instant noodles, soft drinks, and coffee, were found to be significantly associated with the GRS for dyslipidemia. This study also has several limitations. First, the participants comprised an urban-based cohort that included only middle-aged and older adults. In addition, although potential confounding factors that influence dyslipidemia were adjusted for, residual confounding factors that were not included in the analysis may have had an effect. Lastly, the effect of cooking methods and calorie intake were not taken into consideration because SQFFQ does not provide culinary techniques or accurate estimates of calorie intake. Further studies are required to control the potential impact of these limitations on susceptibility to dyslipidemia. 

## 5. Conclusions

This study revealed sex differences in genetic susceptibility to dyslipidemia, which correlated significantly with dietary patterns. Individuals with minor alleles had a higher risk of dyslipidemia compared to those with major alleles of the most significant lead SNPs, although these SNPs differed between males and females, being rs662799 and rs651821. Individuals with a higher GRS had a higher risk of dyslipidemia than those with a lower GRS in both males and females, with higher LDL-C and TG concentrations. Notably, among male participants with high GRSs, a high intake of instant noodles and soft drinks emerged as crucial, whereas high consumption of coffee strongly affected only female participants. These findings support the importance of implementing sex-specific strategic interventions, taking into account genetic predisposition and dietary patterns, to prevent dyslipidemia.

## Figures and Tables

**Table 1:** Demographic characteristics of the study participants.

Characteristics		Male		Female	
		Dyslipidemia *(n* = 4990)	Non-Dyslipidemia (*n* = 10,879)	Dyslipidemia (*n* = 8474)	Non-Dyslipidemia (*n* = 23,219)
Age, years		58.63 ± 0.122	60.29 ± 0.082 ***	58.33 ± 0.079	57.32 ± 0.052 ***
Smoking status	non-smoking	1587 (32.6)	4037 (38)	7784 (92)	21,349 (94.4)
	past smoking	1768 (36.4)	4214 (40)	221 (2.6)	648 (2.9)
	current smoking	1501 (31)	2337 (22) ***	272 (3.2)	595 (2.6) **
Drinking status	non-drinking	1284 (25.7)	2698 (24.8)	5807 (68.6)	15,525 (66.9)
	past drinking	339 (6.8)	793 (7.3)	193 (2.3)	497 (2.1)
	current drinking	3364 (67.4)	7384 (67.9)	2470 (29.1)	7185 (30.9) **
Waist circumference, cm		87.32 ± 0.102	84.29 ± 0.073 ***	80.39 ± 0.087	78.09 ± 0.054 ***
Hip circumference, cm		96.00 ± 0.076	94.49 ± 0.052 ***	93.18 ± 0.062	92.58 ± 0.037 ***
BMI, kg/m^2^		24.96 ± 0.037	23.88 ± 0.026 ***	24.14 ± 0.032	23.33 ± 0.019 ***
Total cholesterol, mg/dL		208.61 ± 0.647	183.99 ± 0.276 ***	235.95 ± 0.449	192.17 ± 0.177 ***
Triglyceride, mg/dL		210.58 ± 1.847	104.75 ± 0.366 ***	164.85 ± 1.030	98.18 ± 0.240 ***
LDL cholesterol, mg/dL		124.90 ± 0.609	105.96 ± 0.260 ***	148.69 ± 0.418	109.74 ± 0.166 ***
HDL cholesterol, mg/dL		45.12 ± 0.192	57.08 ± 0.121 ***	55.61 ± 0.191	62.79 ± 0.091 ***
GRS		16.83 ± 0.042	15.65 ± 0.028 ***	21.18 ± 0.037	20.07 ± 0.022 ***

Values of continuous variables are presented as means and standard error of the mean (SEM), and those of categorical variables are presented as the number of subjects and percentages. Student’s *t*-test and chi-square test were performed as appropriate. Significant differences between dyslipidemia and non-dyslipidemia groups in male and female cohorts separately are indicated by ** *p* < 0.01, *** *p* < 0.001. BMI, body mass index; GRS, genetic risk score.

**Table 2:** Dietary patterns of the study participants.

Parameters		Male		Female	
		Dyslipidemia (*n* = 4990)	Non-Dyslipidemia (*n* = 10,879)	Dyslipidemia (*n* = 8474)	Non-Dyslipidemia (*n* = 23,219)
Pork belly	Low Intake	3619 (72.5)	7976 (73.3)	7117 (84.0)	19,507 (84.0)
	High intake	1371 (27.5)	2903 (26.7)	1357 (16.0)	3712 (16.0)
Beef	Low Intake	3291 (66.0)	7192 (66.1)	5712 (67.4)	15,425 (66.4)
	High Intake	1699 (34.0)	3687 (33.9)	2762 (32.6)	7794 (33.6)
Intestines	Low Intake	4330 (86.8)	9592 (88.2)	5869 (69.3)	16,252 (70.0)
	High Intake	660 (13.2)	1287 (11.8) *	2605 (30.7)	6967 (30.0)
Sausages	Low Intake	3489 (69.9)	8001 (73.5)	6654 (78.5)	17,822 (76.7)
	High Intake	1501 (30.1)	2878 (26.5) ***	1820 (21.48)	5397 (23.2) ***
Chicken	Low Intake	3192 (64.0)	7223 (66.4)	5895 (69.6)	15,696 (67.6)
	High Intake	1798 (36.0)	3656 (33.6) **	2579 (30.4)	7523 (32.4) ***
Soup	Low Intake	3199 (64.1)	7067 (64.9)	5978 (70.5)	16,193 (69.7)
	High Intake	1791 (35.9)	3812 (35.0)	2496 (29.5)	7026 (30.3)
Instant noodles	Low Intake	3488 (69.9)	8129 (74.7)	6358 (75.0)	17,296 (74.5)
	High Intake	1502 (30.1)	2750 (25.2) ***	2116 (25.0)	5923 (25.5)
Snacks	Low Intake	3212 (64.4)	7079 (65.1)	7523 (88.8)	20,420 (88.0)
	High Intake	1778 (35.6)	3800 (34.9)	951 (11.2)	2799 (12.0) *
Soft drinks	Low Intake	3198 (64.1)	7394 (68.0)	6612 (78.0)	18,452 (79.5)
	High Intake	1792 (35.9)	3485 (32.0) ***	1862 (22.0)	4767 (20.5) **
Coffee	Low Intake	3550 (71.1)	8154 (75.0)	7465 (88.0)	20,478 (88.2)
	High Intake	1440 (28.9)	2725 (25.0) ***	1009 (11.9)	2741 (11.8)

Categorical variables are presented as the number of subjects and percentages. A chi-square test was performed. Significant differences between dyslipidemia and non-dyslipidemia groups in male and female cohorts separately are indicated by * *p* < 0.05, ** *p* < 0.01, and *** *p* < 0.001.

**Table 3:** Significant SNPs related to dyslipidemia.

A. Male Participants										
CHR	SNP	POS	Mi	Ma	*p*-Value	Gene	MAF	OR	LB	UB
2	rs13306194	2:21252534_G/A	G	A	1.95 × 10^−08^	*APOB*	0.1043	0.804	0.745	0.8676
2	rs1260326	2:27730940_T/C	T	C	1.10 × 10^−08^	*GCKR*	0.4269	0.8686	0.8277	0.9116
8	rs117026536	8:19818773_G/T	G	T	2.63 × 10^−15^	*LPL*	0.103	0.7364	0.6827	0.7945
8	rs4922120	8:19878598_A/G	A	G	5.31 × 10^−10^	*PTPRD*	0.3089	0.8495	0.8069	0.8944
8	rs2954038	8:126507389_C/A	C	A	9.73 × 10^−12^	*CLCN7*	0.316	1.198	1.138	1.263
11	rs1787701	11:116563992_C/G	G	C	9.29 × 10^−14^	*LGR5*	0.2521	0.814	0.7711	0.8593
11	rs151007118	11:116583864_G/T	T	G	3.90 × 10^−12^	*THADA*	0.1574	1.27	1.187	1.359
11	rs11216118	11:116596395_C/T	T	C	3.11 × 10^−15^	*Consequence none*	0.0783	1.459	1.329	1.603
11	rs180363	11:116597889_T/C	T	C	2.56 × 10^−15^	*Consequence none*	0.154	0.7712	0.7231	0.8224
11	rs61905084	11:116610294_T/C	T	C	3.92 × 10^−23^	*CYP2D6*	0.2248	0.7532	0.7121	0.7966
11	rs113932726	11:116650638_C/T	T	C	7.54 × 10^−41^	*HBB*	0.1074	1.784	1.639	1.942
11	rs662799	11:116663707_G/A	G	A	2.11 × 10^−57^	*APOA5*	0.3543	1.523	1.447	1.604
11	rs11603365	11:117006015_T/A	T	A	1.05 × 10^−11^	*Consequence none*	0.1713	0.8066	0.7581	0.8581
16	rs56156922	16:56987369_T/C	T	C	1.32 × 10^−09^	*CETP*	0.1523	0.8177	0.7662	0.8726
16	rs7499892	16:57006590_C/T	T	C	1.47 × 10^−08^	*CFHR1*	0.1876	1.195	1.124	1.272
19	rs429358	19:45411941_T/C	C	T	3.81 × 10^−12^	*APOE*	0.1126	1.321	1.221	1.43
**B. Female Participants**										
**CHR**	**SNP**	**POS**	**Mi**	**Ma**	***p*** **-Value**	**Gene**	**MAF**	**OR**	**LB**	**UB**
2	rs13306194	2:21252534_G/A	G	A	5.85 × 10^−09^	*APOB*	0.1072	0.8463	0.8001	0.8952
2	rs1260326	2:27730940_T/C	T	C	7.27 × 10^−17^	*GCKR*	0.4256	0.8588	0.8286	0.8900
8	rs2954031	8:126491733_G/T	G	T	9.25 × 10^−11^	*TRIB1*	0.4705	1.124	1.085	1.1650
9	rs9411474	9:136125716_C/G	G	C	1.04 × 10^−08^	*ABO*	0.2464	1.128	1.082	1.1750
9	rs651007	9:136153875_C/T	T	C	3.19 × 10^−08^	*TNFRSF11A*	0.2789	1.118	1.074	1.1620
11	rs11216068	11:116505239_C/T	T	C	4.60 × 10^−09^	*LOC107984372*	0.1207	1.179	1.116	1.2460
11	rs74357270	116523300_A/T	T	A	8.30 × 10^−11^	*LINC02702*	0.296	1.138	1.094	1.1830
11	rs75310100	116578361_G/T	G	T	6.14 × 10^−16^	*HBG2*	0.1975	0.8348	0.799	0.8721
11	rs151007118	116583864_G/T	T	G	1.11 × 10^−15^	*Consequence none*	0.16	1.223	1.164	1.2840
11	rs11216118	116596395_C/T	T	C	2.07 × 10^−18^	*Consequence none*	0.07777	1.356	1.266	1.4510
11	rs180346	116612659_A/C	A	C	7.95 × 10^−29^	*IL18RAP*	0.1936	0.7797	0.7463	0.8146
11	rs1942478	116651463_T/G	T	G	4.15 × 10^−18^	*ZPR1*	0.1927	0.8228	0.7873	0.8598
11	rs2075291	116661392_C/A	A	C	3.38 × 10^−49^	*APOA5*	0.1075	1.575	1.483	1.6730
11	rs651821	11:116662579_C/T	C	T	1.10 × 10^−77^	*APOA5*	0.3584	1.432	1.379	1.4870
11	rs11216183	116781545_C/A	C	A	2.55 × 10^−10^	*SIK3*	0.08296	0.8173	0.7677	0.8700
11	rs143791312	116895355_C/T	C	T	1.15 × 10^−09^	*SIK3*	0.07257	0.8131	0.7607	0.8691
19	rs406315	19:45384116_G/A	A	G	3.92 × 10^−15^	*NECTIN2*	0.2565	0.8832	0.8485	0.9192
19	rs2278426	19:11350488_C/T	C	T	1.16 × 10^−09^	*ANGPTL8*	0.06933	0.7636	0.7139	0.8167
19	rs10119	19:45406673_G/A	A	G	5.01 × 10^−19^	*TOMM40*	0.1193	1.289	1.219	1.3640
19	rs1065853	19:45413233_G/T	G	T	1.11 × 10^−22^	*APOE*	0.04856	0.6736	0.6224	0.7290

CHR, chromosome; SNP, single nucleotide polymorphism; Mi, minor allele; Ma, major allele; MAF, minor allele frequency; OR, odds ratio; LB, lower bound; UB, upper bound. The *p*-value for the OR was adjusted for age and BMI.

**Table 4:** Effects of the minor allele of significant SNPs on dyslipidemia.

	Male			Female		
	rs662799 A > G (APOA5)			rs651821 T > C (APOA5)		
carriers	A:A	A:G	G:G	T:T	T:C	C:C
	(*n* = 7937)	(*n* = 6553)	(*n* = 1379)	(*n* = 15,467)	(*n* = 13,322)	(*n* = 2904)
OR	1	1.506	2.472	1	1.362	2.212
95% CI		1.400–1.619	2.193–2.787		1.290–1.437	2.034–2.407
*p*-value		<0.001	<0.001		<0.001	<0.001

Values are expressed as the OR with 95% CI, which were estimated using logistic regression after adjusting for age and BMI. Carriers of only major alleles were used as the reference group.

**Table 5:** Comparison of biochemical parameters by quartiles of the genetic risk score.

	Male			Female		
	1st Quartile (*n* = 4844)	2nd Quartile (*n* = 6029)	3rd Quartile (*n* = 4996)	1st Quartile (*n* = 9602)	2nd Quartile (*n* = 10,671)	3rd Quartile (*n* = 11,420)
HbA1c, %	5.72 ± 0.012	5.70 ± 0.010	5.68 ± 0.011	5.62 ± 0.007	5.62 ± 0.006	5.63 ± 0.006
λ-GTP, IU/L	40.533 ± 0.7607	42.853 ± 0.7167	45.166 ± 0.9849 ***	21.602 ± 0.1926	22.561 ± 0.2266 *	23.249 ± 0.2442 ***
Albumin, g/dL	4.664 ± 0.0036	4.669 ± 0.0031	4.669 ± 0.0034	4.601 ± 0.0023	4.610 ± 0.022 *	4.610 ± 0.0021 *
AST, IU/L	25.83 ± 0.206	25.92 ± 0.148	26.52 ± 0.260	23.96 ± 0.099	23.91 ± 0.091	24.28 ± 0.121 ^+^
ALP, IU/L	65.71 ± 0.271	66.22 ± 0.291	66.80 ± 0.260 *	68.37 ± 0.200	67.81 ± 0.196	67.49 ± 0.196 **
ALT, IU/L	24.95 ± 0.272	24.82 ± 0.200	25.07 ± 0.222	20.62 ± 0.135	20.41 ± 0.125	20.76 ± 0.152
Creatinine, mg/dL	0.968 ± 0.0044	0.965 ± 0.0038	0.970 ± 0.0050	0.704 ± 0.0016	0.708 ± 0.0017	0.701 ± 0.0017 ^+^
Blood Calcium, mg/dL	9.498 ± 0.0053	9.508 ± 0.0046	9.521 ± 0.0050 **	9.496 ± 0.0038	9.499 ± 0.0037	9.503 ± 0.0035

Values represent means ± SEM from one-way ANOVA with the post hoc Scheffé test. HbA1c, hemoglobin A1c; λ-GTP, gamma-glutamyl transferase; AST, aspartate aminotransferase; ALP, alkaline phosphatase; ALT, alanine transaminase. Significant differences versus the 1st quartile are indicated by * *p* < 0.05, ** *p* < 0.01, and *** *p* < 0.005. Significant differences versus the 2nd quartile are indicated by ^+^ *p* < 0.05.

**Table 6:** Effects of GRS and dietary pattern interactions on dyslipidemia.

A. Meats									
Groups		Male				Female			
		1st Quartile	2nd Quartile	3rd Quartile	*p*-Value	1st Quartile	2nd Quartile	3rd Quartile	*p*-Value
Pork belly	Low Intake	1	1.635 (1.473–1.816)	2.580 (2.321–2.868)	0.907	1	1.352 (1.257–1.454)	2.014 (1.879–2.159)	0.140
	High Intake	1	1.698 (1.433–2.012)	2.699 (2.267–3.213)		1	1.359 (1.149–1.608)	2.250 (1.916–2.642)	
Beef	Low Intake	1	1.642 (1.471–1.832)	2.621 (2.345–2.930)	0.662	1	1.317 (1.214–1.429)	2.019 (1.869–2.182)	0.703
	High Intake	1	1.674 (1.436–1.951)	2.601 (2.228–3.036)		1	1.429 (1.271–1.606)	2.117 (1.891–2.369)	
Intestines	Low Intake	1	1.712 (1.556–1.884)	2.702 (2.451–2.979)	0.370	1	1.333 (1.230–1.444)	2.011 (1.863–2.171)	0.904
	High Intake	1	1.317 (1.033–1.678)	2.118 (1.660–2.702)		1	1.399 (1.239–1.580)	2.137 (1.904–2.399)	
Sausages	Low Intake	1	1.668 (1.500–1.854)	2.706 (2.430–3.013)	0.563	1	1.370 (1.270–1.478)	2.144 (1.966–2.272)	0.213
	High Intake	1	1.619 (1.375–1.908)	2.398 (2.027–2.836)		1	1.301 (1.128–1.499)	1.851 (1.616–2.120)	
Chicken	Low Intake	1	1.718 (1.536–1.921)	2.804 (2.504–3.140)	0.504	1	1.352 (1.248–1.465)	2.035 (1.885–2.197)	0.396
	High Intake	1	1.545 (1.333–1.791)	2.301 (1.979–2.675)		1	1.351 (1.198–1.525)	2.076 (1.850–2.330)	
**B. Soup, instant noodles, snacks, and drinks**									
**Groups**		**Male**				**Female**			
		**1st** **Quartile**	**2nd** **Quartile**	**3rd** **Quartile**	***p*** **-Value**	**1st** **Quartile**	**2nd** **Quartile**	**3rd** **Quartile**	***p*** **-Value**
Soup	Low Intake	1	1.687 (1.508–1.887)	2.770 (2.473–3.102)	0.350	1	1.311 (1.210–1.419)	2.016 (1.868–2.175)	0.738
	High Intake	1	1.599 (1.380–1.852)	2.356 (2.027–2.739)		1	1.452 (1.284–1.641)	2.125 (1.889–2.390)	
Instant noodles	Low Intake	1	1.672 (1.504–1.859)	2.633 (2.366–2.931)	0.028	1	1.384 (1.281–1.494)	2.017 (1.873–2.171)	0.087
	High Intake	1	1.601 (1.357–1.888)	2.569 (2.168–3.044)		1	1.258 (1.098–1.440)	2.154 (1.895–2.449)	
Snacks	Low Intake	1	1.742 (1.558–1.948)	2.820 (2.518–3.159)	0.085	1	1.347 (1.254–1.446)	2.079 (1.943–2.225)	0.210
	High Intake	1	1.504 (1.297–1.744)	2.279 (1.961–2.648)		1	1.414 (1.164–1.716)	1.835 (1.519–217)	
Soft drinks	Low Intake	1	1.739 (1.559–1.940)	2.576 (2.303–2.881)	0.030	1	1.388 (1.287–1.498)	2.117 (1.970–2.276)	0.196
	High Intake	1	1.494 (1.282–1.742)	2.662 (2.282–3.106)		1	1.225 (1.063–1.412)	1.818 (1.586–2.084)	
Coffee	Low Intake	1	1.691 (1.522–1.879)	2.636 (2.368–2.933)	0.188	1	1.338 (1.246–1.437)	2.002 (1.870–2.142)	0.038
	High Intake	1	1.561 (1.320–1.846)	2.560 (2.159–3.034)		1	1.463 (1.203–1.778)	2.489 (2.063–3.003)	

Values represent the adjusted OR, 95% CI, and *p*-value for interaction with GRS. Binary multivariable logistic regression was conducted with the cross-product of the dietary group and GRS quartiles and adjusted for covariates, including age and BMI. The 1st quartile of the GRSs was set as the reference.

## Data Availability

The original data could be available upon request from the Korean Center for Disease Control and Prevention Agency, Republic of Korea.

## Supplementary Materials

The following supporting information can be downloaded at: https://www.mdpi.com/article/10.3390/nu15204385/s1, Supplementary Table S1: Demographic characteristics of the study participants in the entire cohort, Supplementary Table S2: Dietary patterns of the study participants in the entire cohort, Supplementary Table S3: Significant SNPs related to dyslipidemia in the entire cohort, Supplementary Table S4: Comparison of biochemical parameters by quartiles of the genetic risk score in middle-aged, Supplementary Table S5: Comparison of biochemical parameters by quartiles of the genetic risk score in elderly, Supplementary Table S6: Effects of GRS and dietary patterns interactions on dyslipidemia in the entire cohort.
