# Supplementary material for: Associations between the Genetic Heritability of Dyslipidemia and Dietary Patterns in Korean Adults Based on Sex Differences
Source: Nutrients. 2023 Oct 16;15(20):4385. doi: 10.3390/nu15204385 (PMC10609770; doi:10.3390/nu15204385)
Supplement: Supplementary file 1 [file nutrients-15-04385-s001.zip › nutrients-2626322-supplementary.pdf]

Supplementary Table S1. Demographic characteristics of the study participants in the entire cohort.

Characteristic		Entire	
		Dyslipidemia (*n* = 13,464)	Non-dyslipidemia (*n* = 34,098)
Age, years		58.44±0.067	58.27±0.045*
Smoking status	non–smoking	9371(71.4)	25386(76.5)
	past smoking	1989(15.1)	4862(14.7)
	current smoking	1773(13.5)	2932(8.8)***
Drinking status	non–drinking	7091(52.7)	18223(53.5)
	past drinking	532(4.0)	1290(3.8)
	current drinking	5834(43.4)	14569(42.7)
Waist circumference, cm		82.96±0.072	80.07±0.046***
Hip circumference, cm		94.22±0.049	93.19±0.030***
BMI, kg/m^2		24.44±0.025	23.51±0.016***
Total cholesterol, mg/dL		225.81±0.388	189.56±0.151***
Triglyceride, mg/dL		181.79±0.962	100.28±0.201***
LDL cholesterol, mg/dL		140.10±0.360	108.54±0.140***
HDL cholesterol, mg/dL		51.72±0.146	60.97±0.075***
GRS		29.90±0.028	28.76±0.017***

Values of continuous variables are presented as means and standard error of the mean (SEM), and those of categorical variables are presented as the number of subjects and percentages. Student's t-test and chi-square test were performed as appropriate. Significant differences between dyslipidemia and non–dyslipidemia groups in male and female cohorts separately are indicated by * p <0.05, ** p <0.01, and *** p <0.001. BMI, body mass index; GRS, genetic risk score.

Supplementary Table S2. Dietary patterns of the study participants in the entire cohort.

Parameters		Entire	
		**Dyslipidemia (*n* = 13,464)**	**Non-dyslipidemia (*n* = 34,098)**
Pork belly	Low Intake	10555(78.4)	26988(79.1)
	High Intake	2909(21.6)	7110(20.9)
Beef	Low Intake	11847(88.0)	30024(88.1)
	High Intake	1617(12.0)	4074(11.9)
Intestines	Low Intake	9242(68.6)	24053(70.5)
	High Intake	4222(31.4)	10045(29.5)***
Sausages	Low Intake	10143(75.3)	25823(75.7)
	High Intake	3321(24.7)	8275(24.3)
Chicken	Low Intake	9087(67.5)	22918(67.2)
	High Intake	4377(32.5)	11180(32.8)
Soup	Low Intake	10997(81.7)	28015(82.2)
	High Intake	2467(18.3)	6083(17.8)
Instant noodles	Low Intake	10910(81.0)	28253(82.9)
	High Intake	2554(19.0)	5845(17.1)***
Snacks	Low Intake	12075(89.7)	30368(89.1)
	High Intake	1389(10.3)	3730(10.9)*
Soft drink	Low Intake	9810(72.9)	25846(75.8)
	High Intake	3654(27.1)	8252(24.2)***
Coffee	Low Intake	11015(81.8)	28630(84.0)
	High Intake	2449(18.2)	5468(16.0)***

Categorical variables are presented as the number of subjects and percentages. Chi-square test was performed. Significant differences between dyslipidemia and non-dyslipidemia groups in male and female cohorts separately are indicated by $^{*}p$ <0.05, $^{**}p$ <0.01, and $^{***}p$ <0.001.

Supplementary Table S3. Significant SNPs related to dyslipidemia in the entire cohort.

CHR	SNP	POS	Mi	Ma	*p*-value	Gene	MAF	OR	LB	UB
2	rs13306194	2:21252534_G/A	G	A	7.03E–16	APOB	0.1061	0.8305	0.7938	0.8688
2	rs1260326	2:27730940_T/C	T	C	6.26E–24	GCKR	0.4261	0.8628	0.8384	0.8879
8	rs117026536	8:19818773_G/T	G	T	1.05E–15	LPL	0.2776	0.8347	0.7987	0.8724
8	rs2954031	8:126491733_G/T	G	T	9.50E–20	TRIB1	0.1111	1.141	1.109	1.174
8	rs3087723	8:126761402_C/T	C	T	2.93E–08	LOC105375747	0.4747	1.094	1.06	1.13
9	rs9411474	9:136125716_C/G	G	C	6.28E–12	ABO	0.2461	1.123	1.086	1.161
11	rs7946423	11:116503403_A/T	T	A	2.74E–11	LOC107984372	0.1171	1.164	1.113	1.218
11	rs1240772	11:116519129_G/C	C	G	1.28E–08	LINC02702	0.3423	1.091	1.059	1.124
11	rs1145211	11:116557216_A/C	C	A	5.12E–15	*SIDT2*	0.2283	0.875	0.8462	0.9048
11	rs61346349	11:116580798_A/G	A	G	2.08E–08	*Consequence none*	0.09967	0.8752	0.8354	0.917
11	rs117785509	11:116586231_G/A	G	A	6.00E–09	SLC22A4	0.05827	0.8392	0.7911	0.8903
11	rs79408961	11:116588593_C/T	T	C	1.69E–20	PADI4	0.09822	1.26	1.2	1.323
11	rs651821	11:116662579_C/T	C	T	2.49E–132	APOA5	0.3569	1.461	1.417	1.506
11	rs9804646	11:116665079_C/T	C	T	2.66E–29	APOA5	0.1361	0.7937	0.7624	0.8263
11	rs78044162	11:116837089_C/T	C	C	7.99E–20	SIK3	0.1566	0.837	0.8055	0.8696
11	rs4635117	11:116981001_A/G	A	G	1.36E–11	TAGLN	0.2601	1.118	1.083	1.155
11	rs17120523	11:117094591_A/G	A	G	2.72E–11	PCSK7	0.1012	0.8545	0.8159	0.895
16	rs56156922	16:56987369_T/C	T	C	1.49E–10	CETP	0.1594	0.883	0.85	0.9172
16	rs9926440	16:57002663_C/G	C	G	1.23E–09	CETP	0.3268	1.098	1.066	1.132
19	rs2738464	19:11242307_G/C	C	G	7.31E–09	LDLR	0.2755	0.9112	0.8829	0.9404
19	rs737337	19:11347493_T/C	T	C	5.33E–10	DOCK6	0.2653	0.9043	0.876	0.9335
19	rs3112438	19:45359570_A/G	A	G	2.85E–08	NECTIN2	0.0495	0.8343	0.7826	0.8894
19	rs111784051	19:45402262_T/G	T	G	3.50E–22	TOMM40	0.0557	0.7445	0.7013	0.7903
19	rs429358	19:45411941_T/C	C	T	3.48E–29	APOE	0.114	1.301	1.243	1.362
19	rs12709889	19:45453239_G/A	G	A	1.20E–11	APOC2	0.4375	0.9064	0.881	0.9325

CHR, chromosome; SNP, single nucleotide polymorphism; Mi, minor allele; Ma, major allele; MAF, minor allele frequency; OR, odds ratio; LB, lower bound; UB, upper bound. The *p*-value for the OR was adjusted for age and BMI.

Supplementary Table S4. Comparison of biochemical parameters by quartiles of the genetic risk score in middle-aged.

	Male			Female		
	**1st quartile (*n*=2,474)**	**2nd quartile (*n*=3,138)**	**3rd quartile (*n*=2,667)**	**1st quartile (*n*=6,278)**	**2nd quartile (*n*=7,017)**	**3rd quartile (*n*=7,368)**
HbA1c, %	5.634±0.0166	5.605±0.0133	5.609±0.0157	5.609±0.0085	5.601±0.008	5.61±0.0079
λ-GTP, IU/L	44.41±1.145	46.99±1.022	48.44±1.3	22.13±0.281	22.62±0.289	22.4±0.255
Albumin, g/dL	4.719±0.0048	4.721±0.004	4.717±0.0045	4.609±0.0028	4.607±0.0027	4.609±0.0026
AST, IU/L	26.04±0.339	25.82±0.206	26.52±0.323	23.88±0.109	24.08±0.15	23.94±0.143
ALP, IU/L	64.49±0.344	65.22±0.453	65.84±0.348	67.54±0.264	67.93±0.24	67.37±0.238
ALT, IU/L	26.74±0.465	26.19±0.283	26.3±0.32	20.33±0.142	20.88±0.205	20.56±0.18
Creatinine, mg/dL	0.959±0.007	0.954±0.006	0.948±0.003	0.7±0.002	0.704±0.002	0.702±0.002
Blood Calcium, mg/dL	9.535±0.0074	9.553±0.0063	9.55±0.0065	9.497±0.0048	9.5±0.0045	9.5±0.0043

Values represent means ± SEM from one-way ANOVA with the post-hoc Scheffé test. HbA1c, hemoglobin A1c; λ-GTP, gamma-glutamyl transferase; AST, aspartate aminotransferase; ALP, alkaline phosphatase; ALT, alanine transaminase.

Supplementary Table S5. Comparison of biochemical parameters by quartiles of the genetic risk score in elderly.

	Male			Female		
	**1st quartile (*n*=2,370)**	**2nd quartile (*n*=2,891)**	**3rd quartile (*n*=2,329)**	**1st quartile (*n*=3,324)**	**2nd quartile (*n*=3,654)**	**3rd quartile (*n*=4,052)**
HbA1c, %	5.797±0.017	5.796±0.0153	5.771±0.0168	5.646±0.0119	5.661±0.0112	5.639±0.0105
λ-GTP, IU/L	36.49±0.988	38.36±0.995	41.41±1.495*	22.04±0.39	23.62±0.431*	22.57±0.362
Albumin, g/dL	4.607±0.005	4.613±0.0047	4.614±0.0049	4.606±0.0039	4.605±0.0037	4.65±0.0035
AST, IU/L	25.61±0.227	26.02±0.213	26.52±0.418	24.06±0.164	24.4±0.155	24.18±0.167
ALP, IU/L	66.99±0.428	67.3±0.356	67.9±0.39	67.78±0.343	68.98±0.334*	68.21±0.317
ALT, IU/L	23.09±0.267	23.34±0.279	23.65±0.301	20.47±0.231	20.95±0.2	20.46±0.211
Creatinine, mg/dL	0.977±0.006	0.977±0.005	0.995±0.01	0.709±0.003	0.708±0.003	0.71±0.004
Blood Calcium, mg/dL	9.459±0.0074	9.459±0.0065	9.488±0.0077*+	9.498±0.0065	9.505±0.0065	9.5±0.0057

Values represent means ± SEM from one-way ANOVA with the post-hoc Scheffé test. HbA1c, hemoglobin A1c; λ-GTP, gamma-glutamyl transferase; AST, aspartate aminotransferase; ALP, alkaline phosphatase; ALT, alanine transaminase. Significant differences versus 1st quartile at $^{*}p < 0.05$. Significant differences versus 2nd quartile at $^{+}p < 0.05$.

Supplementary Table S6. Effects of GRS and dietary patterns interactions on dyslipidemia in the entire cohort.

**A. Meats**					
**Groups**		**Entire**			
		**1st quartile**	**2nd quartile**	**3rd quartile**	***p*-value**
Pork belly	Low Intake	1	1.469(1.384–1.558)	2.243(2.115–2.378)	0.378
	High Intake	1	1.600(1.429–1.791)	2.306(2.060–2.581)	
Beef	Low Intake	1	1.501(1.419–1.587)	2.295(2.171–2.426)	0.285
	High Intake	1	1.467(1.263–1.704)	2.000(1.723–2.322)	
Intestines	Low Intake	1	1.495(1.404–1.593)	2.207(2.073–2.349)	0.181
	High Intake	1	1.495(1.360–1.644)	2.377(2.164–2.611)	
Sausages	Low Intake	1	1.492(1.404–1.585)	2.282(2.150–2.423)	0.525
	High Intake	1	1.513(1.361–1.682)	2.186(1.966–2.341)	
Chicken	Low Intake	1	1.485(1.393–1.583)	2.271(2.131–2.149)	0.514
	High Intake	1	1.518(1.385–1.664)	2.231(2.037–2.445)	

**B. Soup, instant noodles, snacks, and drinks**					
**Groups**		**Entire**			
		**1st quartile**	**2nd quartile**	**3rd quartile**	***p*-value**
Soup	Low Intake	1	1.529(1.442–1.620)	2.297(2.168–2.433)	0.930
	High Intake	1	1.356(1.200–1.532)	2.089(1.850–2.359)	
Instant noodles	Low Intake	1	1.497(1.412–1.587)	2.271(2.144–2.406)	0.190
	High Intake	1	1.504(1.331–1.699)	2.217(1.962–2.505)	
Snacks	Low Intake	1	1.518(1.436–1.605)	2.302(2.718–2.432)	0.162
	High Intake	1	1.322(1.127–1.550)	1.906(1.625–2.235)	
Soft drink	Low Intake	1	1.456(1.369–1.547)	2.229(2.098–2.367)	< 0.001
	High Intake	1	1.609(1.452–1.782)	2.334(2.106–2.558)	
Coffee	Low Intake	1	1.500(1.416–1.589)	2.244(2.119–2.377)	0.050
	High Intake	1	1.471(1.299–1.666)	2.320(2.050–2.262)	

Values represent the adjusted OR, 95% CI, and *p*-value for interaction with GRS. Binary multivariable logistic regression was conducted with the cross-product of the dietary group and GRS quartiles and adjusted for covariates, including age and BMI. The 1st quartile of the GRS was set as the reference.
